# Association between Hunger and Truancy among Students in Liberia: Analysis of 2017 Global School-Based Student Health Survey

**DOI:** 10.1155/2022/4785238

**Published:** 2022-01-20

**Authors:** Francis Appiah, Tarif Salihu, Yaw Oppong, Henry Yaw Acheampong, Justice Ofosu Darko Fenteng, Andrews Ohene Darteh, Matthew Takyi, Patience Ansomah Ayerakwah, Kingsley Boakye, Edward Kwabena Ameyaw

**Affiliations:** ^1^Department of Population and Health, College of Humanities and Legal Studies, University of Cape Coast, Ghana; ^2^Berekum College of Education, Berekum, Bono Region, Ghana; ^3^Department of Basic Education, Faculty of Education Studies, University of Education, Ghana; ^4^Department of Education Studies, St Monica's College of Education, Mampong-Ashanti Region, Ghana; ^5^Department of Optometry, University of Cape Coast, Ghana; ^6^Department of Epidemiology and Biostatistics, School of Public Health, Kwame Nkrumah University of Science and Technology, Kumasi, Ghana; ^7^L & E Research Consult, Wa, Upper West Region, Ghana

## Abstract

**Background:**

About 83% and 49% of Liberians live beneath the poverty line of US$1.25/day and experience hunger, respectively. Studies have established that hunger has long-term adverse consequence on truancy among students. However, no national level study has investigated contribution of hunger on truancy among in-school students in Liberia. This paper therefore seeks to examine the association between hunger and truancy among students in Liberia. The study hypothesises that there exists a positive association between hunger and truancy.

**Methods:**

This study used the 2017 Liberia Global School-Based Student Health Survey (LGSSHS) and sampled 2,744 students. However, the present study was restricted to 1,613 respondents who had complete information about variable of interest analysed in the study. Hunger and truancy are the main explanatory and outcome variables for this study. At 95% confidence interval, two binary logistic regression models were built with Model I examining relationship between hunger and truancy and Model II controlled for the influence of covariates on truancy. Our findings were reported in odds ratio (OR) and adjusted odds ratio (AOR). All the analysis was done using STATA version 14.0.

**Results:**

Descriptively, 46% were truant, and 65% of students ever experienced hunger. Inferentially, students that ever-encountered hunger had higher odds to truancy (AOR = 1.32, CI = 1.06-1.65). The odds to be truant also increased among those at 15 years and above (AOR = 2.00, CI = 1.46-2.72), who witnessed bullying (AOR = 1.36, CI = 1.10-1.68), that felt lonely (AOR = 1.35, CI = 1.06-1.71), that currently smoke cigarette (AOR = 2.58, CI = 1.64-4.06), and wards whose parents go through their things (AOR = 1.26, CI = 1.03-1.55).

**Conclusions:**

The study concluded that hunger was associated with truancy among students in Liberia. Additionally, students' age, bullying, feeling lonely, cigarette use, and parental concern also determined truancy. Governments, policy makers, and other partners in education should therefore roll out some school-based interventions, such as the school feeding program, which will help minimise the incidence of hunger among students. Such programs should consider the variations in students' background characteristics in its design.

## 1. Introduction

Truancy among students has been identified as a major setback due to its adverse repercussions on the individual, community, and the society at large [1]. Students who skip school are more likely to drop out, have poor academic performance, get dismissed from school, and have a lower chance of graduating [2–4]. Student truancy has been linked to a variety of negative health consequences, including suicidal behavior, substance abuse (alcohol, nicotine, and marijuana), criminality, and delinquent behavior [5–8]. Within 30-day period, truancy rates range from 21.6% in Swaziland [9], 36.6% in Mozambique [10], to 58.8% in Zambia [10] .

Globally, studies have discovered several enablers of truancy among students especially in Africa. Individual characteristics such as skipping hunger, being a man, growing older, and being in the upper school level, for example, have been identified as facilitators of truancy among pupils [7, 11, 12]. Several mental and behavioral characteristics have also been found to predict truant conduct in students in several researches. For instance, students who go through depression, anxiety, or partake in substance use are more likely to be truant relative to those without such features [7, 10, 13–15]. Other predictors include being bullied, injured, gang-related violence, physical attack, poor school environment, uninteresting classwork, strained student-teacher relationship, lack of school connectedness, and fighting within the school milieu [11, 16–20].

In Liberia, food insecurity has long been recognized as a growing social, economic, and public health issue after the civil war that occurred between 1989 and 2003 [21]. Since 2003, the country has been wrestling with its hostile past while making efforts to advance a strategy for the future [21]. Food insecurity is predominant especially in isolated areas of the country where poor road networks are prevalent. Majority of Liberians (83%) live beneath the poverty line of US$1.25/day and 49% of the populace experience hunger [22]. The country depends largely on imported foodstuff because of low agricultural productivity fuelled by poor farming practices, high post-harvest losses, and poor road networks [22]. Hunger has been proven to have a long-term impact on truancy among students in various cross-sectional studies in high-income nations [23–25]. Place of residence, belonging to family of poor quintile, having female household head, parent education level, and food insecurity were the possible factors for hunger among adolescent and students [26]. According to Shankar et al. [27], there is a significant correlation between hunger and truancy among students. In addition, a study conducted by Bernal et al. [28] found that students who experienced hunger were more likely to skip school (truancy) in Venezuela's Miranda State.

Conversely, students who are provided with breakfast while in school and/or lunch have high school turnout, academic performance, and good attitude in classrooms [29–31]. It has also been shown that intellectually, students with food have augmented focus and studying ability [32]. Nevertheless, in low- and middle-income countries (LMICs), particularly Liberia, the link between hunger and truancy has received little attention. Despite the fact that food insecurity/hunger has the ability to impair students' attendance and performance, no research on the link between hunger and truancy among Liberian students has been conducted. Investigating the association between hunger and truancy among Liberian students will be a critical step in developing and implementing effective truancy prevention educational interventions. This paper therefore is aimed at investigating the relationship that exist between hunger and truancy among Liberian students. The study hypothesises that there exists a positive association between hunger and truancy.

## 2. Methods

### 2.1. Study Design and Data Source

The study adopted a cross-sectional survey design and used data from the 2017 Liberia Global School-Based Student Health Survey (LGSSHS). The survey was conducted among students between grades 7 and 12. The survey among other indicators assessed students' views and experiences with alcohol, tobacco, drugs, eating pattern, personal hygiene and mental health, physical fitness, injuries, and sexuality attitudes. With Liberia Ministry of Education serving as the lead organization for the survey, the survey had financial assistance from World Health Organization and the US Centers for Disease Control and Prevention.

The survey adopted a two-stage cluster sampling technique. The first stage involved choosing schools based on a probability proportional to the number of pupils enrolled. Following that, a random selection of classes was made, making every student in those classes eligible to participate in the study. In all, 2,744 students took part in the survey. However, this study was restricted to 1,613 respondents who had complete information about the variables of interest to this study. On a computer scannable answer forms, students self-reported their answers to each question. The survey achieved a school response rate of 98%, and the student response rate was 73%. The survey's overall response rate was 71%. Details about the entire methodology are available in the World Health Organization Report for LGSSHS [33].

### 2.2. Derivation of Outcome Variable

The dependent variable for the study was “truancy,” which was determined by asking, “How many days did you miss courses or school without permission in the last 30 days?” accompanied by these responses: “0 days,” “1 or 2 days,” “3-5 days,” “6-9 days,” and “10 or more days.” Following Henry (2010) and Gastic (2008) definition of truancy, thus absenting from school or class as indicated in earlier study by Seidu [10], study participants who affirmed “0 days” were classified as “never been truant” while the rest were classified as “ever been truant.” “Never been truant” was coded as “0,” and “ever been truant” was also coded as “1.”

### 2.3. Derivation of Independent Variable

Hunger was the primary independent variable in this study. This was obtained from the question, “How often did you go hungry because there was not enough food in your home in the last 30 days?” and the responses were “never,” “rarely,” “sometime,” “most of the time,” and “always.” These were dichotomised where “no” denoted students who mentioned that they never experienced hunger and “yes” for the rest who experienced hunger irrespective of the frequency of hunger. Finally, “no” and “yes” responses were assigned “0” and “1,” respectively.

Twelve covariates were added to the analysis: sex, age, grade, bullied, attempted suicide, felt lonely, could not sleep, current cigarette use, have close friends, parents check homework, parents know about free time, and parents go through their things. These variables were not chosen apriori, but they have been shown to influence truancy [7, 9, 34, 35]. To make the results easily readable, age was recoded as “11-14 years” and “15 years and above”; grade recoded as “8-10 grade” and “11 and above grade”; bullied recoded into “no” and “yes”; attempted suicide recoded into “no” and “yes”; felt lonely recoded into “no” and “yes”; could not sleep recoded into “no” and “yes”; current cigarette use recoded as “no” and “yes”; have close friends recoded as “no” and “yes”; parents check homework recoded as “no” and “yes”; parents know about free time recoded as “no” and “yes”; and finally whether parents or guardians look through their belongings without their permission or otherwise recoded as “no” and “yes.”

### 2.4. Statistical Analysis

In this study, we hypothesised a positive association between hunger and truancy. Following this assumption, we calculated the students who were truant or otherwise. Thereafter, we performed univariable descriptive computation of the independent variable and the covariates. We further did a bivariable descriptive computation of hunger and the covariates across truancy. Additionally, we employed a chi-square test of independence to assess the relationship between the outcome variable and the independent variables, and a cut-off point was set at 0.05. As such, any independent variable that could not meet the cut-off point was not entered into the multivariate model. Subsequently, at 95% confidence interval and 5% alpha threshold, we built two binary logistic regression models whereby Model I examined the relationship between hunger and truancy only and Model II catered for the influence of the covariates on truancy.

Our findings were reported in odds ratio (OR) and adjusted odds ratio (aOR). An odds above 1 was explained as increased students' likelihood to be truant, and an odds below 1 meant otherwise. We applied the weighting factor inherent in the dataset to cater for sampling errors while “vif” command was applied to test for multicollinearity between our independent variables. Our independent variables showed no signs of multicollinearity (mean VIF = 1.09; maximum VIF = 1.15; minimum VIF = 1.02) (Appendix 1). We also made use of the “linktest” command to do model specification diagnosis, and the results indicated that our regression model was well specified. We carried out all analysis using STATA version 14.0.

### 2.5. Ethical Considerations

In this study, we depended on already existing dataset; as such, we were not directly involved in the ethical considerations applicable to research involving human participation. We obtained the dataset through WHO website, and the data is opened to the public at https://extranet.who.int/ncdsmicrodata/index.php/catalog/646/get_microdata.

## 3. Results

### 3.1. Hunger, Socio-Demographic Characteristics, and Truancy in Liberia

Generally, it was found that 46% of them were truant while a little above half (54%) were not truant (data not shown).  Table 1 is the descriptive results on hunger, socio-demographics, and truancy in Liberia. It was evident that 65% had ever experienced hunger. The analysis revealed that 54% of the students were males, 84% were in Grade 15 or higher, and 53% experienced bullying. Over two-thirds (70%) indicated that they have not attempted suicide whereas 69% proclaimed that they have ever felt lonely. Over two in three persons (74%) mentioned that they could not sleep while nine out of ten (93%) declared that they were not smoking cigarette.

Eighty-nine percent had close friends; 79% reported that parents check their homework while 81% disclosed that parents know about their free time. Also, 59% mentioned that parents go through their things. Finally, from the chi-square test of independence, it was ascertained that with the exception of sex, grade, have close friends, parents check homework, and parents know their free time, rest of the independent variables had an association with truancy (see Table 1).

### 3.2. Inferential Results for the Study

Table 2 presents the binary logistic regression results of hunger and truancy. Students who had ever encountered hunger had higher odds to be truant (OR = 1.55, CI = 1.25-1.91), and after controlling for the selected covariates, this observation remained the same (AOR = 1.32, CI = 1.06-1.65). Those at aged 15 and above had higher odds to be truant compared with those at age 11-14 (AOR = 2.00, CI = 1.46-2.72) just as among those who had witnessed bullying compared to those who had not (AOR = 1.36, CI = 1.10-1.68).

Students who felt lonely were more inclined to truancy as compared to their peers who had not felt lonely (AOR = 1.35, CI = 1.06-1.71). Moreover, those who were currently smoking cigarette were over two-fold probable to be truant as opposed those who had not smoked cigarette (AOR = 2.58, CI = 1.64-4.06). Additionally, students whose parents go through their things were more probable to be truant compared to those whose parents do not (AOR = 1.26, CI = 1.03-1.55). Considering the model diagnostic testing, it was clear that the model had been well-defined (Table 2).

## 4. Discussion

The main thrust for this study was to assess the relationship between hunger and truancy among students in Liberia. Descriptively, the study revealed that the prevalence of truancy among Liberian students was 46%. Pengpid and Peltzer [7] observed varying prevalence of truancy across selected Asian countries ranging from 15% in Vietnam to 28% in Malaysia. These are generally lower than what we found in Liberia. A study in Mozambique also reported a prevalence of 36.6% [34]. The variation in truancy observed in this study compared to findings from other countries could be attributed to the different school climates in which students find themselves and also partly due to the contextual variation in study settings.

The key finding was that students who were hungry were more likely to be absent from school. This corresponds with a study by Komakech and Osuu [36] who identified that hunger was a reason why students absented themselves from school in Uganda. It also confirms what was found in other previous studies [7, 9, 10, 37]. Two main plausible explanations have been offered in explaining the effects of hunger on truancy. Most students who go hungry, according to Seidu et al. [10], come from impoverished families and, as a result, may miss school due to their work engagement at home. On the other hand, Wadesango and Machingambi [38] have suggested that students who go hungry have a low socioeconomic status and that work on part-time basis to make ends meet. Other covariates that significantly influenced truancy among students were students' age, bullying, feeling lonely, cigarette use, and parental concern. Although these determinants are not novel for truancy among students, however, they still remain as motivators to truancy as reported in previous studies [7, 9, 34].

The present study showed that students aged 15 years or more were shown to be more likely to be truant than those aged 11 to 14. Similarly, Seidu [10] found that students aged 15 and above were more likely to be truant compared to those aged 11-14 in Mozambique. The result is in consonance with a study by Muula et al. [37] who found that adolescents who fall within age 14 and below are less likely to be truant compared to those who are 15 years and above in Zambia. Muula et al. [37] explained that younger students are more likely to be under parental supervision than relatively older pupils and may thus be less likely to be truant than older pupils. Relatedly, it was found that students who reported being victims of bullying were more likely to be truants. Seidu et al. [10] and Peltzer and Pengpid [7] explained that adolescents who have experienced bullying victimization may miss school in order to escape further victimization by their peers. It is therefore important for schools' antibullying policies to reduce bullying in schools [37, 39].

In agreement to previous studies [37, 40], the current study found that students who felt lonely were inclined towards truancy. Muula et al. [37] and Henry and Huizinga [40] indicated that students who felt lonely had higher tendency to be truants and students with delinquent peers stand a higher chance of being truants. As evidenced in previous studies [9, 10, 41], adolescents who smoked and used tobacco were more likely to be truants. In Mozambique, it was found that adolescents who engaged in smoking and tobacco usage had higher odds of truancy [34]. A plausible explanation could be that truant students are less monitored and often unchecked at home and as such have free will to smoke, take alcohol, and engage in related deviant behaviors [7]. In light of the foregoing, the current study discovered that students whose parents checked their homework were less likely to be truants than those whose parents did not. This result reechoes what some scholars have reported in earlier researches [7, 37, 42]. They observed that the propensity for students to be truants declines among those whose parents supervise their assignments and home works.

### 4.1. Strengths and Weaknesses

The study is novel and first of its kind to have investigated the association of hunger on truancy among students aged 13-17 in Liberia. The findings and conclusions were drawn from a nationally representative survey dataset hence present the views of in-school adolescents in Liberia. Also, the larger sample size and refined analytical processes render the findings robust. However, the cross-sectional nature of the survey implies that causal relationship cannot be established with the dataset used. Also, the survey failed to capture the views of out of school adolescents. Social desirability bias cannot be overlooked in this study. Other important predictors that might influence the association between hunger and truancy were not captured by LGSSHS such as family income, participation in family income generating activities, parent education level, and parent marital status. Also, the strength of association between hunger and truancy may be mis-estimated in the study because the response rate was only 71.0%, and it was most likely contributed by students who were absent or play truant during data collection.

## 5. Conclusion

The rate of truancy among Liberian students was found to be relatively high. Noticeably, hunger was strongly associated with truancy. Additionally, age, bullying, feeling lonely, cigarette use, and parental concern affected truancy in Liberia. Government, policy makers, and other partners in education should therefore roll out some school-based interventions, such as the school feeding program, which will help minimise the incidence of hunger among students. Loneliness, which happens to be one of the predisposing factors of truancy, can be managed through effective guidance and counselling in schools. School authorities on the other hand should address the issues of bullying and the use of cigarette through the enforcement of school rules and regulations to aid in the reduction of truancy. Additionally, parents should be encouraged during Parent Teachers' Association (PTA) meetings to show love and concern in their wards' educational life.

## Figures and Tables

**Table 1:** Hunger, socio-demographic characteristics, and truancy in Liberia (*N* = 1,613).

Variable	Weighted (*N*)	Weighted (%)	Truancy		*X* ^2^ (*p* value)
			No (%)	Yes (%)	
Hunger					16.641 (0.001)
Never	561	35	62	38	
Ever	1052	65	52	48	
Sex					1.712 (0.191)
Male	864	54	54	46	
Female	749	46	57	43	
Age (in years)					27.510 (0.001)
11-14	263	16	71	29	
15 and above	1350	84	53	47	
Grade					0.244 (0.621)
8-10	1262	78	55	45	
11 and above	351	22	56	44	
Bullied					20.498 (0.001)
No	850	53	60	40	
Yes	763	47	49	51	
Attempted suicide					19.553 (0.001)
No	1125	70	59	41	
Yes	488	30	47	53	
Felt lonely					18.109 (0.001)
No	419	31	63	37	
Yes	1194	69	52	48	
Could not sleep					9.528 (0.002)
No	416	26	62	38	
Yes	1156	74	53	47	
Current cigarette use					33.953 (0.001)
No	1497	93	57	43	
Yes	116	7	28	72	
Have close friends					0.072 (0.789)
No	176	11	56	44	
Yes	1437	89	55	45	
Parents check homework					0.047 (0.828)
No	345	21	55	45	
Yes	1267	79	55	45	
Parents know about free time					1.153 (0.283)
No	312	19	58	42	
Yes	1301	81	55	45	
Parents go through their things					9.654 (0.002)
No	665	41	60	40	
Yes	948	59	52	48	

Source: 2017 LGSSHS.

**Table 2:** Binary logistic regression on hunger and truancy.

Variable	Model I		Model II	
	OR	95% CI	AOR	95% CI
Hunger				
Never	Ref	1.1	Ref	1.1
Ever	1.55^∗∗∗^	(1.25-1.91)	1.32^∗∗^	(1.06-1.65)
Age				
11-14			Ref	1.1
15 and above			2.00^∗∗∗^	(1.46-2.72)
Bullied				
No			Ref	1.1
Yes			1.36^∗∗^	(1.10-1.68)
Attempted suicide				
No			Ref	1.1
Yes			1.21	(0.96-1.53)
Felt lonely				
No			Ref	1.1
Yes			1.35^∗^	(1.06-1.71)
Could not sleep				
No			Ref	1.1
Yes			1.07	(0.83-1.37)
Current cigarette use				
No			Ref	1.1
Yes			2.58^∗∗∗^	(1.64-4.06)
Parents go through their things				
No			Ref	1.1
Yes			1.26^∗^	(1.03-1.55)
Linktest				
_hat			1.00^∗∗∗^	(0.79-1.22)
_hatsq			0.01	(-0.23-0.25)

Sources: LGSSHS 2017. OR: odds ratio; AOR: adjusted odds ratio; CI: confidence interval in square brackets; Ref: reference category. ^∗^*p* < 0.05, ^∗∗^*p* < 0.01, ^∗∗∗^*p* < 0.001.

## Data Availability

The dataset is freely accessible at https://extranet.who.int/ncdsmicrodata/index.php/catalog/646/get_microdata.

## Supplementary Materials

Supplementary Materials The supplementary file is a multicollinearity test results. The multicollinearity with variance inflation factor (vif) output is used to determine intercorrelations among the predictor variables in a regression model. A threshold of 10 was set as highly correlated while 1 indicated no correlation. However, our independent variables showed no signs of multicollinearity (mean VIF = 1.09; maximum VIF = 1.15; minimum VIF = 1.02).
