# Supplementary material for: Association between Hunger and Truancy among Students in Liberia: Analysis of 2017 Global School-Based Student Health Survey
Source: Biomed Res Int. 2022 Jan 20;2022:4785238. doi: 10.1155/2022/4785238 (PMC8794671; doi:10.1155/2022/4785238)
Supplement: Supplementary Materials — The supplementary file is a multicollinearity test results. The multicollinearity with variance inflation factor (vif) output is used to determine intercorrelations among the predictor variables in a regression model. A threshold of 10 was set as highly correlated while 1 indicated no correlation. However, our independent variables showed no signs of multicollinearity (mean VIF = 1.09; maximum VIF = 1.15; minimum VIF = 1.02). [file 4785238.f1.docx]

**Manuscript ID: 4785238**

**Appendix 1: Multicollinearity test results**

| Variable | VIF | 1/VIF |
| --- | --- | --- |
| Suicide | 1.15 | 0.871739 |
| Could not sleep | 1.13 | 0.882484 |
| Felt lonely | 1.13 | 0.888347 |
| Current cigarette use | 1.10 | 0.907143 |
| Bullied | 1.10 | 0.912847 |
| Hunger | 1.07 | 0.938741 |
| Age | 1.04 | 0.963351 |
| Parents go through their things | 1.02 | 0.977684 |
| Mean VIF | 1.09 | |
